# Dreaming of wetscapes: Waking to the realities of restoration

**DOI:** 10.1007/s13280-023-01956-8

**Published:** 2023-12-08

**Authors:** James Douglas Langston, Daniel Steven Mendham, Niken Sakuntaladewi

**Affiliations:** 1 CSIRO Environment, Building 101, Clunies Ross Street, Black Mountain, ACT 2601 Australia; 2 Research Center for Ecology and Ethnobiology, National Research and Innovation Agency (BRIN), Jalan Raya Jakarta-Bogor km 46, Cibinong, 16911 Indonesia

Comment to: Temmink, R.J., B.J. Robroek, G. van Dijk, A.H. Koks, S.A. Käärmelahti, A. Barthelmes, M.J. Wassen, R. Ziegler, et al. 2023. Wetscapes: Restoring and maintaining peatland landscapes for sustainable futures. *Ambio* 52: 1519–1528. 10.1007/s13280-023-01875-8. 

## Introduction

Temmink et al. (2023) champion the case for restoring the world’s peatlands to concomitantly address shared climate and nature goals. We share this view and consider that restoration of the worlds peatlands is critical for the future of people and nature. Degradation of tropical peatlands is correlated with precarious human development; it reduces human lifespan and increases vulnerability, including through increased fires and floods, as well as having lasting and significant economic and environmental costs. While Temmink et al. (2023) promote desirable ‘wetscape’ designs based on a sound biophysical understanding of peatlands, we think their approach overlooks the complex social, cultural, and political dynamics shaping peatlands. We offer this commentary in response to Temmink et al. (2023) so that restoration can be effective, efficient, and just for those communities who are living and working on peatlands. These communities are already highly vulnerable and can become even more vulnerable if change is foisted upon them without due care and consideration of their needs.

We, as part of a larger group of researchers engaged in Indonesia’s peatland restoration,^1^ agree that peatlands deserve greater share of attention on the world stage. Indonesia’s threatened peatlands (covering 13.4 Mha) store 30% more carbon than the biomass of all Indonesian forests (covering 94.1 Mha), and are the source of 30–60% of Indonesia’s annual greenhouse gas emissions. Increased attention is not sufficient. There are more degraded peatlands than wet and intact ones, and in 2023 fire incidences are increasing. We are troubled by naïve recommendations on how peatlands should be spatially configured to deliver the ecosystem services necessary to mitigate and adapt to climate change. Wicked problems are the root cause of hindered restoration efforts.

Many have already recognized the need for better zoning on peatlands in ways that allow for diversity in livelihoods and management scenarios (Applegate et al. 2022). Local people are the key to unlocking the restoration effort. But for too long, local peoples’ aspirations have come at the expense of global visions prioritizing public goods values of nature. High-income nations may have the luxury of affording this approach, but it does not align with realities faced by many communities at the ground level, where food, shelter and culture take precedence (Jalilov et al., in press). In this commentary we (1) identify limits to Temmink’s proposal for achieving wetscapes. We then (2) highlight a more inclusive approach to supporting sustainable and equitable peatlands in Indonesia. We think this approach is more fit-for-purpose. We call for more robust theories of change based on the actors who value peatlands, how they value them, and we encourage learning for governance and justice in situ.

## Dreaming of wetscapes

### The science of restoration

Better spatial plans, prescriptive livelihood options, and heuristics will not solve the problems. Science, experts, and practitioners must move past the temptation for grand designs (Sayer et al. 2008). Restoration problems will only be solved by moving into the politically difficult realms of human behavior and the value systems that underlie land-use change. Evidence shows that impact is more likely when science pivots towards embedded, transdisciplinary, and de-colonized endeavors. This involves co-creating transition pathways that focus on justice. The search for sustainable and equitable outcomes for both people and nature in peatlands hinges on engaging with how nature is valued, and by whom (Pascual et al. 2023). This science requires dealing head-on with context specificity, bureaucratic inertia, and political economies characterized by power asymmetries (Langston et al. 2019; Purnomo et al. 2021).

Peatlands are frontiers of development in Indonesia and the tropics more broadly. Local people are highly dependent on the cash economy and are among the least powerful actors affecting peatland restoration governance. Contexts for restoration are rapidly changing. Temmink et al. (2023) point out that “… socio-economic conditions and hydrological constraints hitherto prevent rewetting and restoration on large scale, which calls for rethinking landscape use.” However, their proposed solutions overlook the complex drivers of degradation, and therefore could cause more harm.

### Into complex contestation

Conserving the remaining intact peatlands in Indonesia indeed requires restoration in addition to protection. Boundaries are leaky, so a whole of hydrological unit approach is desirable, with the end goal resembling a spatially optimized wetscape. But context is everything, and hydrological units fit within broader social-ecological contexts (Clarke and Rieley 2019). Figure 2 of Temmink et al. (2023) elucidates a seemingly straightforward decision-tree to help achieve a multifunctional wetscape (Hiller and Fisher 2023). However in reality each decision is deeply contested, and this is non-trivial. The first decision to make, “*is productive use necessary*?” According to who? Local communities, Indigenous peoples, distant decision-makers, national and international issue-advocates may all have different responses. The multiple and often conflicting preferences of these groups will manifest in diverse definitions of what constitutes a ‘productive use’ of nature.

The subsequent question in the decision-tree “*is rewetting possible, if yes then rewet*”, is equally problematic. Possible according to who? At what cost? The choice of metrics can drastically affect how rewet the landscape is both reported and becomes. Indonesian government metrics include quantity of boreholes drilled (for fire suppression), groundwater monitoring stations installed, rather than by complete closure of canals and persistently high or above ground water levels. Who is responsible for rewetting? How are local communities accounted for in this equation? They are the most at risk people in the decision-tree and are further disempowered by the decisions being made by others. Should scientists be the cause of their increased vulnerability? Each point in the decision-tree is hotly contested by a plurality of decision-makers.

## Waking to restoration

### Value-centric theories of change

Any wise use of peatlands (Joosten and Clarke 2002) must be built on more rigorous theories of change, to support contextualized roadmaps. A healthy and well-functioning peatland must be co-defined by local people and the range of actors driving peatland management outcomes. Theories of change must grapple with the underlying drivers land-use change and the contested value systems affecting peatland health (Abdurrahim et al. 2023). Reductively, restoration and degradation are two sides of a value coin. Restoration projects and top down initiatives have been stymied by the multi-dimensional value of nature, as held by diverse groups of people (Abdurrahim et al. 2023). In this sense, restoration and degradation are different views of a multi-faceted dice, depending on the actor’s vantage point. Pursuing common ground on what ‘restoration’ means, how nature is valued and by whom, and installing robust learning mechanisms are vital to the robustness of any spatial plan (Rawluk et al. 2022; Robins et al. 2022). Current economic systems and development aspirations often mean degraded peatlands are more highly ‘valued’ than restored peatlands; this is a fundamental driver of degradation to be addressed (Sakuntaladewi et al. 2022). Therefore, inclusive and transparent value-setting processes are crucial to developing a legitimate and comprehensive understanding of the ‘costs’ of different land-uses. Theoretical, or ‘fictive’, carbon values of preserved peatlands are of little value to decision-makers who prioritize cash-in-hand from estate crops, mining, or other land-uses that are incompatible with rewet, intact peatlands (Lestari et al. 2021; Yuwati et al. 2021; Abdurrahim et al. 2023). Our research shows that paludiculture is generally an undesirable option for local communities already living in precarity on Indonesia’s frontier (Mendham et al., in press). Livelihood options that do not exceed the opportunity cost of land-based livelihoods (including oil palm and rubber), will risk making local people more vulnerable and violate peoples’ rights to self-determination. Delaying prosperity delays any likelihood of long-term restoration success. For peatland restoration to succeed, the full range of actors interested in transitioning to rewet peatlands need to navigate these pluralistic and often irreconcilable values.

### Governing peatlands

Preconditions to effective spatial planning include good governance, a topic well scrutinized in Indonesia (Sayer et al. 2021). Improvements in procedural and distributive justice must shift the peatland decision-making paradigm. Tenure is still a politically sensitive topic in Indonesia, but imperative to good peatland governance. How tenure is fit for purpose is a major question in peatland restoration efforts. Transitions to rewet peatlands must include mapping a clear pathway to reward local people for restoration so that the most vulnerable are not further disadvantaged. Since the *International Peatland Society* published their wise use guidelines and frameworks (Joosten and Clarke 2002), scientific advances have regularly tried to confront governance issues. Latest frameworks build on visions to better reconcile peatlands’ environmental, social and economic functions whilst respecting their local, regional and global values (Clarke and Rieley 2019). Concerted efforts to do this, and to embed learning at peatland’scape scale in situ is needed (Sari et al. 2021).

In summary, we concur with Temmink et al. (2023) that peatland restoration is critical for the future of our planet. But as we have outlined above, the decisions that need to be taken at various levels are not as straight forward or clear cut as they appear on paper. Right now peatland situations are precarious, in part due to visions of restoration that get in the way of local peoples’ agency and prosperity, prolonging meaningful restoration. Solutions need to be co-created with local communities, not for or to them. Sustainable and equitable pathways to wetscapes can only realistically arise from coalitions seeking institutional mechanisms that prioritize justice and empowerment.

## Footnotes

1 We represent a group of more than 80 scientists, researchers (and practitioners) committed to the restoration of Indonesia’s peatlands (*previously under the program moniker* *Gambut Kita*, *Indonesian for ‘Our Peatlands’*).
